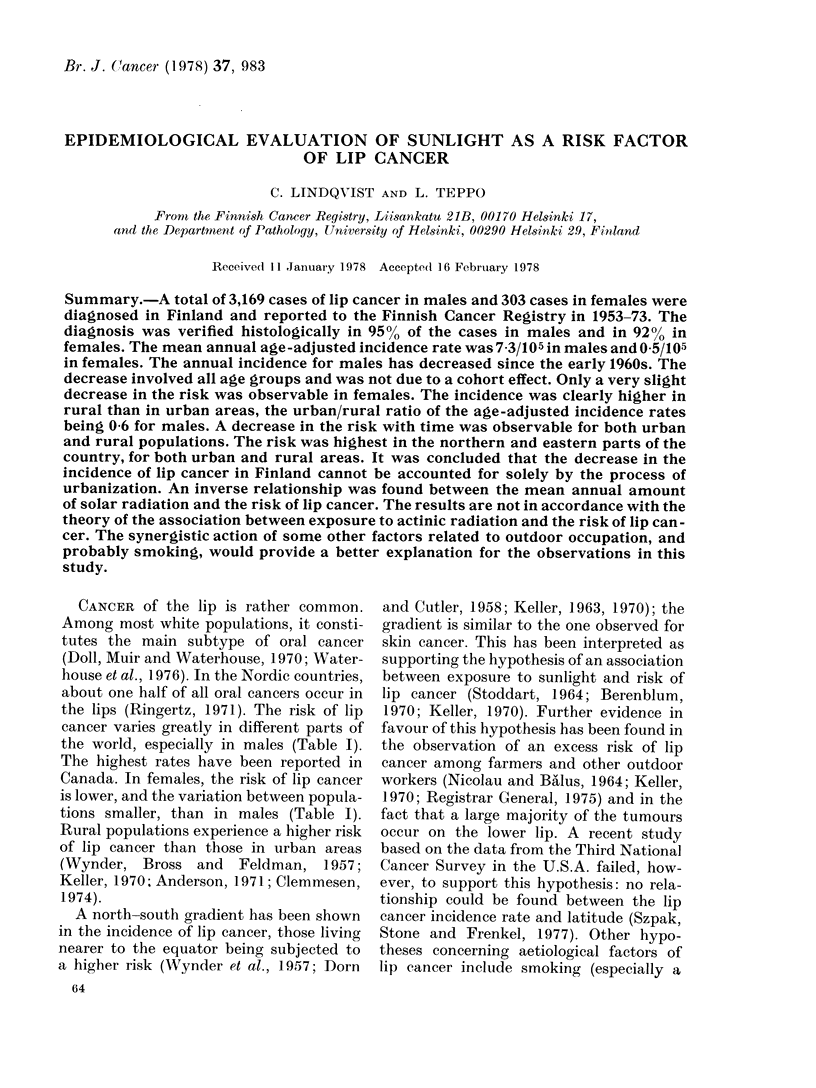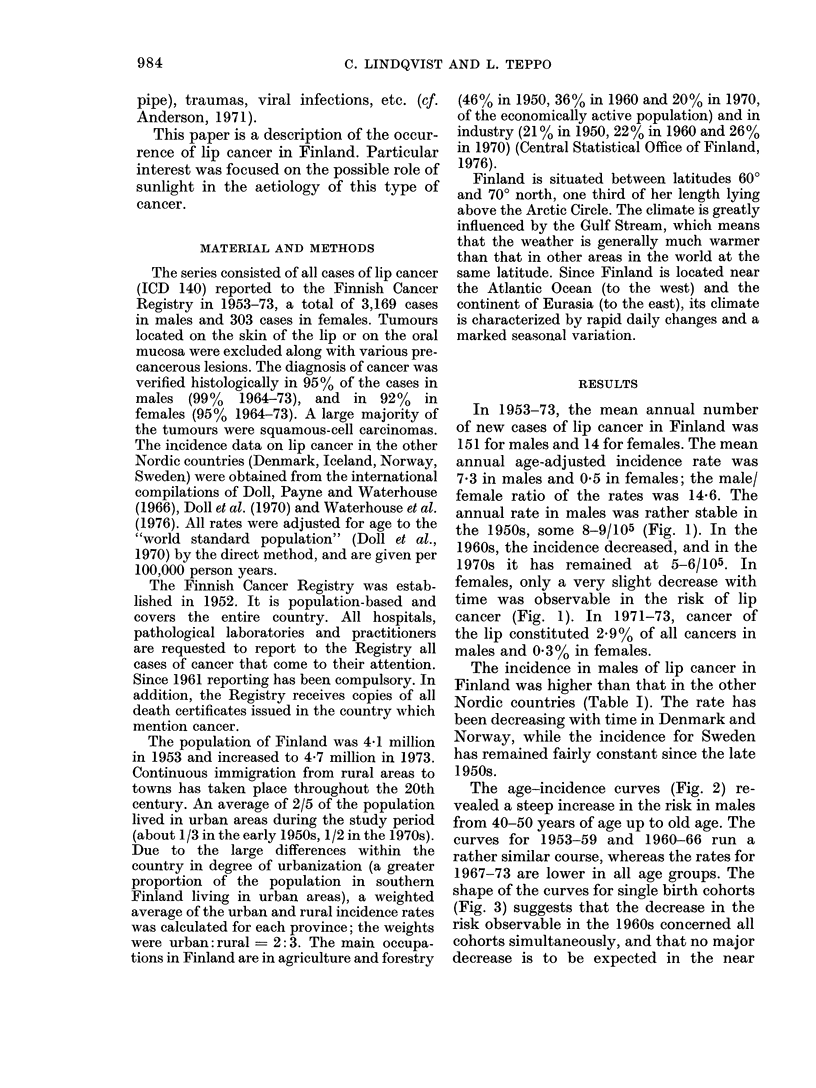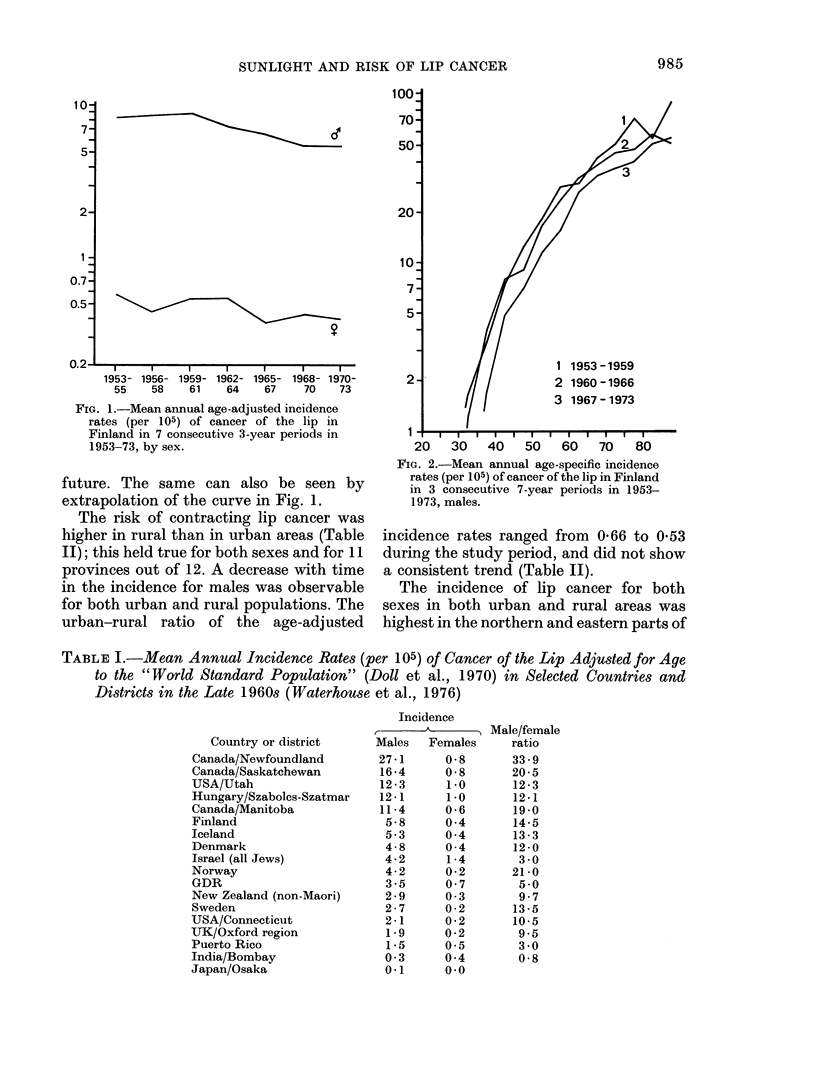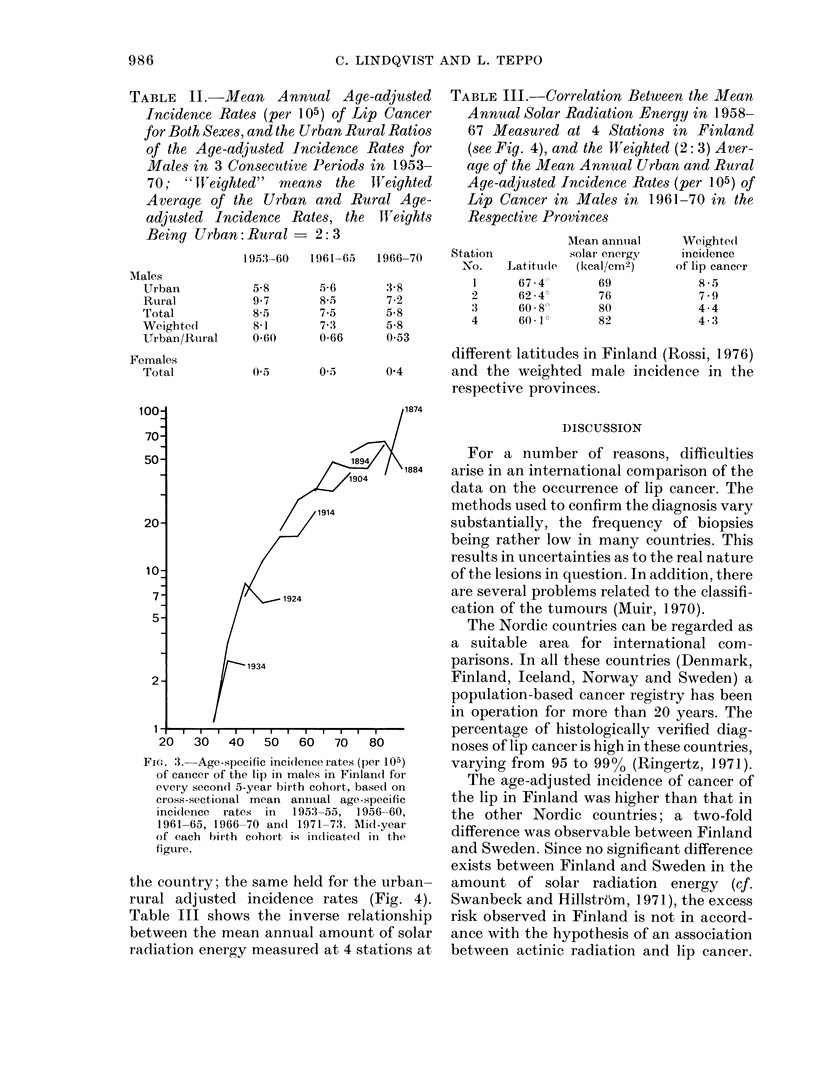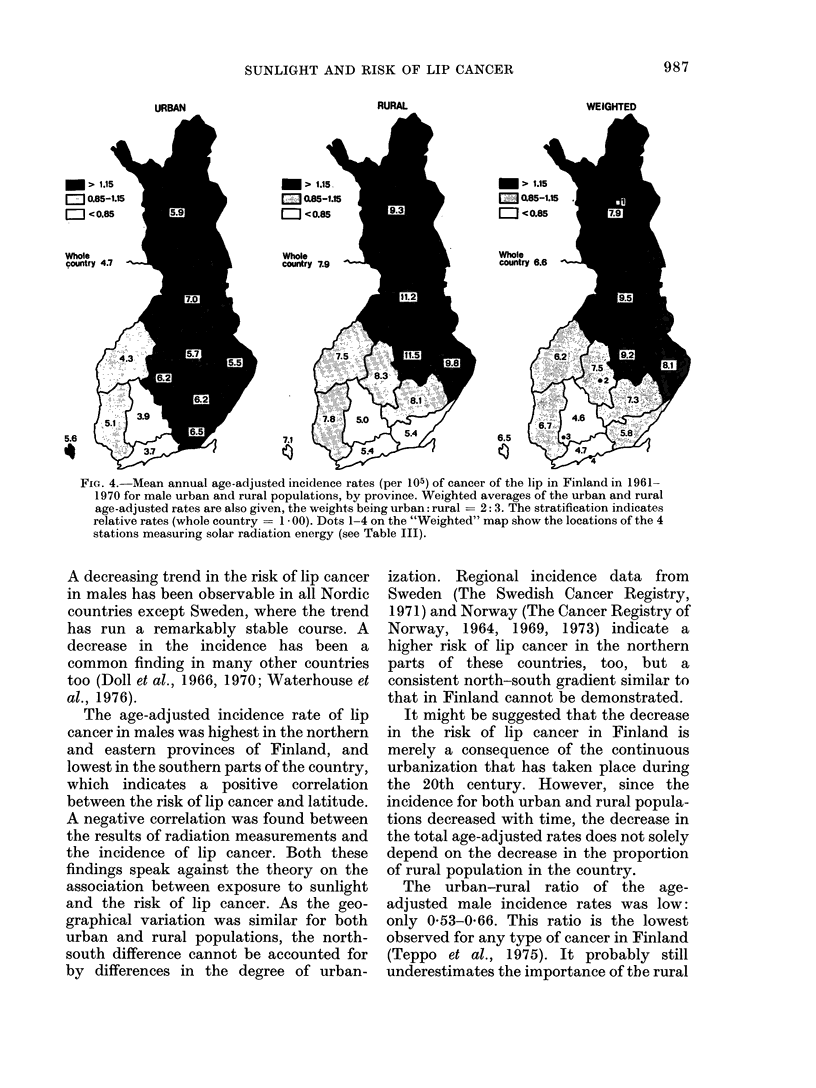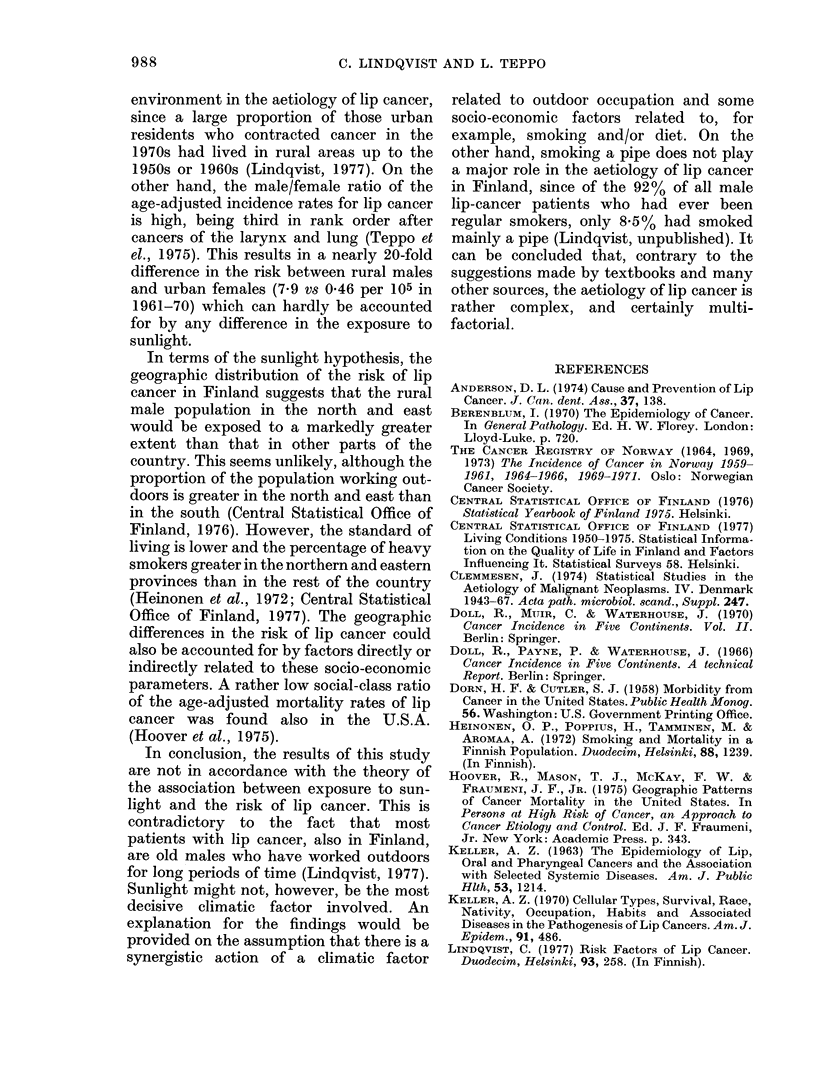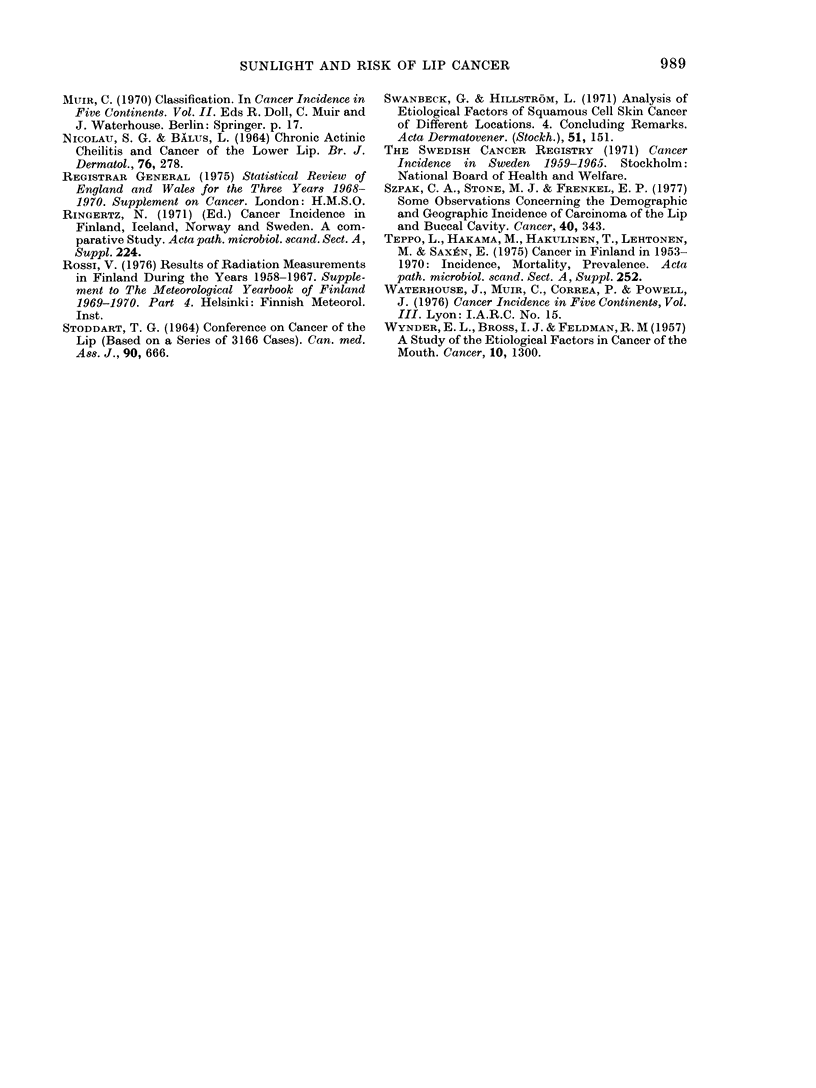# Epidemiological Evaluation of Sunlight as a Risk Factor of Lip Cancer

**DOI:** 10.1038/bjc.1978.143

**Published:** 1978-06

**Authors:** C. Lindqvist, L. Teppo

## Abstract

A total of 3,169 cases of lip cancer in males and 303 cases in females were diagnosed in Finland and reported to the Finnish Cancer Registry in 1953-73. The diagnosis was verified histologically in 95% of the cases in males and in 92% in females. The mean annual age-adjusted incidence rate was 7·3/10^5^ in males and 0·5/10^5^ in females. The annual incidence for males has decreased since the early 1960s. The decrease involved all age groups and was not due to a cohort effect. Only a very slight decrease in the risk was observable in females. The incidence was clearly higher in rural than in urban areas, the urban/rural ratio of the age-adjusted incidence rates being 0·6 for males. A decrease in the risk with time was observable for both urban and rural populations. The risk was highest in the northern and eastern parts of the country, for both urban and rural areas. It was concluded that the decrease in the incidence of lip cancer in Finland cannot be accounted for solely by the process of urbanization. An inverse relationship was found between the mean annual amount of solar radiation and the risk of lip cancer. The results are not in accordance with the theory of the association between exposure to actinic radiation and the risk of lip cancer. The synergistic action of some other factors related to outdoor occupation, and probably smoking, would provide a better explanation for the observations in this study.


Br. J. Cancer (1978) 37, 983

### EPIDEMIOLOGICAL EVALUATION OF SUNLIGHT AS A RISK FACTOR OF LIP CANCER

C. LINDQVIST AND L. TEPPO

*From the Finnish Cancer Registry, Liisankatu 21B, 00170 Helsinki 17, and the Department of Pathology, University of Helsinki, 00290 Helsinki 29, Finland*

Received 11 January 1978 Accepted 16 February 1978

**Summary.—A total of 3,169 cases of lip cancer in males and 303 cases in females were diagnosed in Finland and reported to the Finnish Cancer Registry in 1953–73. The diagnosis was verified histologically in 95% of the cases in males and in 92% in females. The mean annual age-adjusted incidence rate was 7·3/10$^5$ in males and 0·5/10$^5$ in females. The annual incidence for males has decreased since the early 1960s. The decrease involved all age groups and was not due to a cohort effect. Only a very slight decrease in the risk was observable in females. The incidence was clearly higher in rural than in urban areas, the urban/rural ratio of the age-adjusted incidence rates being 0·6 for males. A decrease in the risk with time was observable for both urban and rural populations. The risk was highest in the northern and eastern parts of the country, for both urban and rural areas. It was concluded that the decrease in the incidence of lip cancer in Finland cannot be accounted for solely by the process of urbanization. An inverse relationship was found between the mean annual amount of solar radiation and the risk of lip cancer. The results are not in accordance with the theory of the association between exposure to actinic radiation and the risk of lip cancer. The synergistic action of some other factors related to outdoor occupation, and probably smoking, would provide a better explanation for the observations in this study.**

CANCER of the lip is rather common. Among most white populations, it constitutes the main subtype of oral cancer (Doll, Muir and Waterhouse, 1970; Waterhouse *et al.*, 1976). In the Nordic countries, about one half of all oral cancers occur in the lips (Ringertz, 1971). The risk of lip cancer varies greatly in different parts of the world, especially in males (Table I). The highest rates have been reported in Canada. In females, the risk of lip cancer is lower, and the variation between populations smaller, than in males (Table I). Rural populations experience a higher risk of lip cancer than those in urban areas (Wynder, Bross and Feldman, 1957; Keller, 1970; Anderson, 1971; Clemmesen, 1974).

A north–south gradient has been shown in the incidence of lip cancer, those living nearer to the equator being subjected to a higher risk (Wynder *et al.*, 1957; Dorn and Cutler, 1958; Keller, 1963, 1970); the gradient is similar to the one observed for skin cancer. This has been interpreted as supporting the hypothesis of an association between exposure to sunlight and risk of lip cancer (Stoddart, 1964; Berenblum, 1970; Keller, 1970). Further evidence in favour of this hypothesis has been found in the observation of an excess risk of lip cancer among farmers and other outdoor workers (Nicolau and Bălus, 1964; Keller, 1970; Registrar General, 1975) and in the fact that a large majority of the tumours occur on the lower lip. A recent study based on the data from the Third National Cancer Survey in the U.S.A. failed, however, to support this hypothesis: no relationship could be found between the lip cancer incidence rate and latitude (Szpak, Stone and Frenkel, 1977). Other hypotheses concerning aetiological factors of lip cancer include smoking (especially a pipe), traumas, viral infections, etc. (*cf.* Anderson, 1971).

This paper is a description of the occurrence of lip cancer in Finland. Particular interest was focused on the possible role of sunlight in the aetiology of this type of cancer.

### MATERIAL AND METHODS

The series consisted of all cases of lip cancer (ICD 140) reported to the Finnish Cancer Registry in 1953–73, a total of 3,169 cases in males and 303 cases in females. Tumours located on the skin of the lip or on the oral mucosa were excluded along with various precancerous lesions. The diagnosis of cancer was verified histologically in 95% of the cases in males (99% 1964–73), and in 92% in females (95% 1964–73). A large majority of the tumours were squamous-cell carcinomas. The incidence data on lip cancer in the other Nordic countries (Denmark, Iceland, Norway, Sweden) were obtained from the international compilations of Doll, Payne and Waterhouse (1966), Doll *et al.* (1970) and Waterhouse *et al.* (1976). All rates were adjusted for age to the "world standard population" (Doll *et al.*, 1970) by the direct method, and are given per 100,000 person years.

The Finnish Cancer Registry was established in 1952. It is population-based and covers the entire country. All hospitals, pathological laboratories and practitioners are requested to report to the Registry all cases of cancer that come to their attention. Since 1961 reporting has been compulsory. In addition, the Registry receives copies of all death certificates issued in the country which mention cancer.

The population of Finland was 4·1 million in 1953 and increased to 4·7 million in 1973. Continuous immigration from rural areas to towns has taken place throughout the 20th century. An average of 2/5 of the population lived in urban areas during the study period (about 1/3 in the early 1950s, 1/2 in the 1970s). Due to the large differences within the country in degree of urbanization (a greater proportion of the population in southern Finland living in urban areas), a weighted average of the urban and rural incidence rates was calculated for each province; the weights were urban:rural = 2:3. The main occupations in Finland are in agriculture and forestry (46% in 1950, 36% in 1960 and 20% in 1970, of the economically active population) and in industry (21% in 1950, 22% in 1960 and 26% in 1970) (Central Statistical Office of Finland, 1976).

Finland is situated between latitudes 60° and 70° north, one third of her length lying above the Arctic Circle. The climate is greatly influenced by the Gulf Stream, which means that the weather is generally much warmer than that in other areas in the world at the same latitude. Since Finland is located near the Atlantic Ocean (to the west) and the continent of Eurasia (to the east), its climate is characterized by rapid daily changes and a marked seasonal variation.

### RESULTS

In 1953–73, the mean annual number of new cases of lip cancer in Finland was 151 for males and 14 for females. The mean annual age-adjusted incidence rate was 7·3 in males and 0·5 in females; the male/female ratio of the rates was 14·6. The annual rate in males was rather stable in the 1950s, some 8–9/$10^5$ (Fig. 1). In the 1960s, the incidence decreased, and in the 1970s it has remained at 5–6/$10^5$. In females, only a very slight decrease with time was observable in the risk of lip cancer (Fig. 1). In 1971–73, cancer of the lip constituted 2·9% of all cancers in males and 0·3% in females.

The incidence in males of lip cancer in Finland was higher than that in the other Nordic countries (Table I). The rate has been decreasing with time in Denmark and Norway, while the incidence for Sweden has remained fairly constant since the late 1950s.

The age–incidence curves (Fig. 2) revealed a steep increase in the risk in males from 40–50 years of age up to old age. The curves for 1953–59 and 1960–66 run a rather similar course, whereas the rates for 1967–73 are lower in all age groups. The shape of the curves for single birth cohorts (Fig. 3) suggests that the decrease in the risk observable in the 1960s concerned all cohorts simultaneously, and that no major decrease is to be expected in the near

FIG. 1.—Mean annual age-adjusted incidence rates (per $10^5$) of cancer of the lip in Finland in 7 consecutive 3-year periods in 1953–73, by sex.

FIG. 2.—Mean annual age-specific incidence rates (per $10^5$) of cancer of the lip in Finland in 3 consecutive 7-year periods in 1953–1973, males.

future. The same can also be seen by extrapolation of the curve in Fig. 1.

The risk of contracting lip cancer was higher in rural than in urban areas (Table II); this held true for both sexes and for 11 provinces out of 12. A decrease with time in the incidence for males was observable for both urban and rural populations. The urban–rural ratio of the age-adjusted incidence rates ranged from 0·66 to 0·53 during the study period, and did not show a consistent trend (Table II).

The incidence of lip cancer for both sexes in both urban and rural areas was highest in the northern and eastern parts of

TABLE I.—*Mean Annual Incidence Rates* (*per* $10^5$) *of Cancer of the Lip Adjusted for Age to the "World Standard Population"* (*Doll* et al., 1970) *in Selected Countries and Districts in the Late* 1960*s* (*Waterhouse* et al., 1976)

| Country or district | Incidence: Males | Incidence: Females | Male/female ratio |
|---|---|---|---|
| Canada/Newfoundland | 27·1 | 0·8 | 33·9 |
| Canada/Saskatchewan | 16·4 | 0·8 | 20·5 |
| USA/Utah | 12·3 | 1·0 | 12·3 |
| Hungary/Szabolcs-Szatmar | 12·1 | 1·0 | 12·1 |
| Canada/Manitoba | 11·4 | 0·6 | 19·0 |
| Finland | 5·8 | 0·4 | 14·5 |
| Iceland | 5·3 | 0·4 | 13·3 |
| Denmark | 4·8 | 0·4 | 12·0 |
| Israel (all Jews) | 4·2 | 1·4 | 3·0 |
| Norway | 4·2 | 0·2 | 21·0 |
| GDR | 3·5 | 0·7 | 5·0 |
| New Zealand (non-Maori) | 2·9 | 0·3 | 9·7 |
| Sweden | 2·7 | 0·2 | 13·5 |
| USA/Connecticut | 2·1 | 0·2 | 10·5 |
| UK/Oxford region | 1·9 | 0·2 | 9·5 |
| Puerto Rico | 1·5 | 0·5 | 3·0 |
| India/Bombay | 0·3 | 0·4 | 0·8 |
| Japan/Osaka | 0·1 | 0·0 | |

TABLE II.—*Mean Annual Age-adjusted Incidence Rates (per* $10^5$*) of Lip Cancer for Both Sexes, and the Urban Rural Ratios of the Age-adjusted Incidence Rates for Males in* 3 *Consecutive Periods in* 1953–70; *"Weighted" means the Weighted Average of the Urban and Rural Age-adjusted Incidence Rates, the Weights Being Urban: Rural* = 2 : 3

| | 1953–60 | 1961–65 | 1966–70 |
|---|---|---|---|
| Males | | | |
| Urban | 5·8 | 5·6 | 3·8 |
| Rural | 9·7 | 8·5 | 7·2 |
| Total | 8·5 | 7·5 | 5·8 |
| Weighted | 8·1 | 7·3 | 5·8 |
| Urban/Rural | 0·60 | 0·66 | 0·53 |
| Females | | | |
| Total | 0·5 | 0·5 | 0·4 |

FIG. 3.—Age-specific incidence rates (per $10^5$) of cancer of the lip in males in Finland for every second 5-year birth cohort, based on cross-sectional mean annual age-specific incidence rates in 1953–55, 1956–60, 1961–65, 1966–70 and 1971–73. Mid-year of each birth cohort is indicated in the figure.

the country; the same held for the urban–rural adjusted incidence rates (Fig. 4). Table III shows the inverse relationship between the mean annual amount of solar radiation energy measured at 4 stations at

TABLE III.—*Correlation Between the Mean Annual Solar Radiation Energy in* 1958–67 *Measured at* 4 *Stations in Finland (see Fig.* 4), *and the Weighted* (2 : 3) *Average of the Mean Annual Urban and Rural Age-adjusted Incidence Rates (per* $10^5$*) of Lip Cancer in Males in* 1961–70 *in the Respective Provinces*

| Station No. | Latitude | Mean annual solar energy (kcal/cm$^2$) | Weighted incidence of lip cancer |
|---|---|---|---|
| 1 | 67·4° | 69 | 8·5 |
| 2 | 62·4° | 76 | 7·9 |
| 3 | 60·8° | 80 | 4·4 |
| 4 | 60·1° | 82 | 4·3 |

different latitudes in Finland (Rossi, 1976) and the weighted male incidence in the respective provinces.

### DISCUSSION

For a number of reasons, difficulties arise in an international comparison of the data on the occurrence of lip cancer. The methods used to confirm the diagnosis vary substantially, the frequency of biopsies being rather low in many countries. This results in uncertainties as to the real nature of the lesions in question. In addition, there are several problems related to the classification of the tumours (Muir, 1970).

The Nordic countries can be regarded as a suitable area for international comparisons. In all these countries (Denmark, Finland, Iceland, Norway and Sweden) a population-based cancer registry has been in operation for more than 20 years. The percentage of histologically verified diagnoses of lip cancer is high in these countries, varying from 95 to 99% (Ringertz, 1971).

The age-adjusted incidence of cancer of the lip in Finland was higher than that in the other Nordic countries; a two-fold difference was observable between Finland and Sweden. Since no significant difference exists between Finland and Sweden in the amount of solar radiation energy (*cf.* Swanbeck and Hillström, 1971), the excess risk observed in Finland is not in accordance with the hypothesis of an association between actinic radiation and lip cancer.

FIG. 4.—Mean annual age-adjusted incidence rates (per $10^5$) of cancer of the lip in Finland in 1961–1970 for male urban and rural populations, by province. Weighted averages of the urban and rural age-adjusted rates are also given, the weights being urban : rural = 2 : 3. The stratification indicates relative rates (whole country = 1·00). Dots 1–4 on the "Weighted" map show the locations of the 4 stations measuring solar radiation energy (see Table III).

A decreasing trend in the risk of lip cancer in males has been observable in all Nordic countries except Sweden, where the trend has run a remarkably stable course. A decrease in the incidence has been a common finding in many other countries too (Doll *et al.*, 1966, 1970; Waterhouse *et al.*, 1976).

The age-adjusted incidence rate of lip cancer in males was highest in the northern and eastern provinces of Finland, and lowest in the southern parts of the country, which indicates a positive correlation between the risk of lip cancer and latitude. A negative correlation was found between the results of radiation measurements and the incidence of lip cancer. Both these findings speak against the theory on the association between exposure to sunlight and the risk of lip cancer. As the geographical variation was similar for both urban and rural populations, the north-south difference cannot be accounted for by differences in the degree of urbanization. Regional incidence data from Sweden (The Swedish Cancer Registry, 1971) and Norway (The Cancer Registry of Norway, 1964, 1969, 1973) indicate a higher risk of lip cancer in the northern parts of these countries, too, but a consistent north–south gradient similar to that in Finland cannot be demonstrated.

It might be suggested that the decrease in the risk of lip cancer in Finland is merely a consequence of the continuous urbanization that has taken place during the 20th century. However, since the incidence for both urban and rural populations decreased with time, the decrease in the total age-adjusted rates does not solely depend on the decrease in the proportion of rural population in the country.

The urban–rural ratio of the age-adjusted male incidence rates was low: only 0·53–0·66. This ratio is the lowest observed for any type of cancer in Finland (Teppo *et al.*, 1975). It probably still underestimates the importance of the rural environment in the aetiology of lip cancer, since a large proportion of those urban residents who contracted cancer in the 1970s had lived in rural areas up to the 1950s or 1960s (Lindqvist, 1977). On the other hand, the male/female ratio of the age-adjusted incidence rates for lip cancer is high, being third in rank order after cancers of the larynx and lung (Teppo *et el.*, 1975). This results in a nearly 20-fold difference in the risk between rural males and urban females (7·9 *vs* 0·46 per $10^5$ in 1961–70) which can hardly be accounted for by any difference in the exposure to sunlight.

In terms of the sunlight hypothesis, the geographic distribution of the risk of lip cancer in Finland suggests that the rural male population in the north and east would be exposed to a markedly greater extent than that in other parts of the country. This seems unlikely, although the proportion of the population working outdoors is greater in the north and east than in the south (Central Statistical Office of Finland, 1976). However, the standard of living is lower and the percentage of heavy smokers greater in the northern and eastern provinces than in the rest of the country (Heinonen *et al.*, 1972; Central Statistical Office of Finland, 1977). The geographic differences in the risk of lip cancer could also be accounted for by factors directly or indirectly related to these socio-economic parameters. A rather low social-class ratio of the age-adjusted mortality rates of lip cancer was found also in the U.S.A. (Hoover *et al.*, 1975).

In conclusion, the results of this study are not in accordance with the theory of the association between exposure to sunlight and the risk of lip cancer. This is contradictory to the fact that most patients with lip cancer, also in Finland, are old males who have worked outdoors for long periods of time (Lindqvist, 1977). Sunlight might not, however, be the most decisive climatic factor involved. An explanation for the findings would be provided on the assumption that there is a synergistic action of a climatic factor related to outdoor occupation and some socio-economic factors related to, for example, smoking and/or diet. On the other hand, smoking a pipe does not play a major role in the aetiology of lip cancer in Finland, since of the 92% of all male lip-cancer patients who had ever been regular smokers, only 8·5% had smoked mainly a pipe (Lindqvist, unpublished). It can be concluded that, contrary to the suggestions made by textbooks and many other sources, the aetiology of lip cancer is rather complex, and certainly multifactorial.

### REFERENCES

Anderson, D. L. (1974) Cause and Prevention of Lip Cancer. *J. Can. dent. Ass.*, **37**, 138.

Berenblum, I. (1970) The Epidemiology of Cancer. In *General Pathology*. Ed. H. W. Florey. London: Lloyd-Luke. p. 720.

The Cancer Registry of Norway (1964, 1969, 1973) *The Incidence of Cancer in Norway 1959–1961, 1964–1966, 1969–1971.* Oslo: Norwegian Cancer Society.

Central Statistical Office of Finland (1976) *Statistical Yearbook of Finland 1975.* Helsinki.

Central Statistical Office of Finland (1977) Living Conditions 1950–1975. Statistical Information on the Quality of Life in Finland and Factors Influencing It. Statistical Surveys 58. Helsinki.

Clemmesen, J. (1974) Statistical Studies in the Aetiology of Malignant Neoplasms. IV. Denmark 1943–67. *Acta path. microbiol. scand., Suppl.* **247.**

Doll, R., Muir, C. & Waterhouse, J. (1970) *Cancer Incidence in Five Continents. Vol. II.* Berlin: Springer.

Doll, R., Payne, P. & Waterhouse, J. (1966) *Cancer Incidence in Five Continents. A technical Report.* Berlin: Springer.

Dorn, H. F. & Cutler, S. J. (1958) Morbidity from Cancer in the United States. *Public Health Monog.* **56.** Washington: U.S. Government Printing Office.

Heinonen, O. P., Poppius, H., Tamminen, M. & Aromaa, A. (1972) Smoking and Mortality in a Finnish Population. *Duodecim, Helsinki,* **88,** 1239. (In Finnish).

Hoover, R., Mason, T. J., McKay, F. W. & Fraumeni, J. F., Jr. (1975) Geographic Patterns of Cancer Mortality in the United States. In *Persons at High Risk of Cancer, an Approach to Cancer Etiology and Control.* Ed. J. F. Fraumeni, Jr. New York: Academic Press. p. 343.

Keller, A. Z. (1963) The Epidemiology of Lip, Oral and Pharyngeal Cancers and the Association with Selected Systemic Diseases. *Am. J. Public Hlth,* **53,** 1214.

Keller, A. Z. (1970) Cellular Types, Survival, Race, Nativity, Occupation, Habits and Associated Diseases in the Pathogenesis of Lip Cancers. *Am. J. Epidem.,* **91,** 486.

Lindqvist, C. (1977) Risk Factors of Lip Cancer. *Duodecim, Helsinki,* **93,** 258. (In Finnish).

MUIR, C. (1970) Classification. In *Cancer Incidence in Five Continents. Vol. II.* Eds R. Doll, C. Muir and J. Waterhouse. Berlin: Springer. p. 17.

NICOLAU, S. G. & BĂLUS, L. (1964) Chronic Actinic Cheilitis and Cancer of the Lower Lip. *Br. J. Dermatol.*, **76**, 278.

REGISTRAR GENERAL (1975) *Statistical Review of England and Wales for the Three Years 1968–1970. Supplement on Cancer.* London: H.M.S.O.

RINGERTZ, N. (1971) (Ed.) Cancer Incidence in Finland, Iceland, Norway and Sweden. A comparative Study. *Acta path. microbiol. scand. Sect. A, Suppl.* **224.**

ROSSI, V. (1976) Results of Radiation Measurements in Finland During the Years 1958–1967. *Supplement to The Meteorological Yearbook of Finland 1969–1970. Part 4.* Helsinki: Finnish Meteorol. Inst.

STODDART, T. G. (1964) Conference on Cancer of the Lip (Based on a Series of 3166 Cases). *Can. med. Ass. J.*, **90**, 666.

SWANBECK, G. & HILLSTRÖM, L. (1971) Analysis of Etiological Factors of Squamous Cell Skin Cancer of Different Locations. 4. Concluding Remarks. *Acta Dermatovener. (Stockh.)*, **51**, 151.

THE SWEDISH CANCER REGISTRY (1971) *Cancer Incidence in Sweden 1959–1965.* Stockholm: National Board of Health and Welfare.

SZPAK, C. A., STONE, M. J. & FRENKEL, E. P. (1977) Some Observations Concerning the Demographic and Geographic Incidence of Carcinoma of the Lip and Buccal Cavity. *Cancer*, **40**, 343.

TEPPO, L., HAKAMA, M., HAKULINEN, T., LEHTONEN, M. & SAXÉN, E. (1975) Cancer in Finland in 1953–1970: Incidence, Mortality, Prevalence. *Acta path. microbiol. scand. Sect. A, Suppl.* **252.**

WATERHOUSE, J., MUIR, C., CORREA, P. & POWELL, J. (1976) *Cancer Incidence in Five Continents, Vol. III.* Lyon: I.A.R.C. No. 15.

WYNDER, E. L., BROSS, I. J. & FELDMAN, R. M (1957) A Study of the Etiological Factors in Cancer of the Mouth. *Cancer*, **10**, 1300.